# Discovery of Novel Sources of Vitamin B_12_ in Traditional Korean Foods from Nutritional Surveys of Centenarians

**DOI:** 10.1155/2010/374897

**Published:** 2011-03-08

**Authors:** Chung Shil Kwak, Mee Sook Lee, Se In Oh, Sang Chul Park

**Affiliations:** ^1^Institute on Aging, Seoul National University, Seoul 110-810, Republic of Korea; ^2^Department of Food and Nutrition, Hannam University, Daejeon 305-811, Republic of Korea; ^3^Department of Food and Nutrition, Seoil University, Seoul 131-702, Republic of Korea; ^4^Department of Biochemistry and Molecular Biology, Aging and Apoptosis Research Center, Seoul National University, Seoul 110-799, Republic of Korea

## Abstract

Human longevity can be explained by a variety of factors, among them, nutritional factor would play an important role. In our study of Korean centenarians for their longevity, the apparent nutritional imbalance in the traditional semi-vegetarian diet raised a special attention, especially on vitamin B_12_ status, supplied by animal foods. Interestingly, we found that the prevalence of vitamin B_12_ deficient Korean centenarians was not higher compared with those from Western nations with animal-oriented traditional foods. We assumed that there might be some unveiled sources for vitamin B_12_ in the Korean traditional foods. Screening of vitamin B_12_ contents has revealed that some traditional soybean-fermented foods, such as *Doenjang* and *Chunggukjang*, and seaweeds contain considerable amounts of vitamin B_12_. Taken together, it can be summarized that the traditional foods, especially of fermentation, might be evaluated for compensation of the nutritional imbalance in the vegetable-oriented dietary pattern by supplying vitamin B_12_, resulting in maintenance of health status.

## 1. Introduction

It is well known that older adults comprise the fastest growing portion of the world population and that the oldest old (including centenarians) are one of the fastest growing subgroups. The oldest population varies greatly depending upon nation, region, and biodemographic trends. At the end of the 20th century, it was reported that the centenarian population numbered approximately 1 per 100,000 persons, with higher numbers (10 per 100,000) in developed countries, and still higher numbers in the regions with very low mortality levels, such as Okinawa prefecture, in southern Japan (about 34 per 100,000) while about 4.7per 100,000 existed in Korea during this period [1].

Korean centenarian numbers were first reported to be 2,220 (172 males and 2,048 females) in the year 2000, based on the birth record data from Statistics Korea, and the ratio of centenarians to the elderly of 65 and older was reported to be 6.6% [2]. However, we have found that one third of birth records of older people may be mistaken due to problems within the civil registration system and therefore we produced a more conservative estimate of 1,481 Korean centenarians in the year 2000 [3]. Since that time, the National Bureau of Statistics of Korea has not officially reported the number of centenarians. The actual number of Korean centenarians is still waiting to be confirmed after individual age verification can take place.

When the gender difference in number of centenarians is taken into consideration, female centenarians are found to far outnumber male centenarians all over the world, except for some limited areas such as Sardinia, Western China, or the Middle East, the latter two of which are likely unreliable due to a lack of documentation and/or lack of a long-standing civil registration system [4]. However, the ratio of female centenarians to male centenarians in Korea was reported to be the highest among nations at 11.5 females for every male in 2000 [2]. The exact reasons for these gender differences have yet to be elucidated; however, they are likely due to a combination of social, biological, and demographical factors. Moreover, our observations indicate that the gender gap seems to be closing in the recent years. Korean life expectancy has improved considerably during the post war period and reached 76.5 years for men and 83.3 years for women, with the older population (aging 65 and older) reaching 10.7% in 2009 [5].

**Figure 1:**
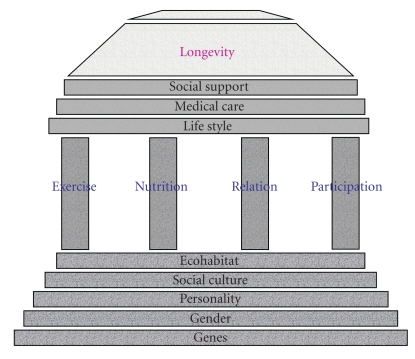
Park's Temple Model of Human Longevity.

In order to study human longevity and its related factors in a scientific manner, the analysis of the relative influence (and interaction) of a variety of variables may be necessary. For integration of these variables, we propose a new model for human longevity, which might be named “Park's Temple Model for Human Longevity” (Figure 1). The premise of this model is based on the concept that human longevity could be compared to building up a temple, consisting of 3 essential components as bottom, pillars, and roof top. For building up a temple, all the components should be strengthened and balanced for safety and stability. The bottom components of the temple are basically fixative or not readily changeable variables, such as genetics, gender, personality, ecology, social structures, or cultures. The pillar components of the temple, related with personal life styles or health behaviors, might be readily modifiable variables and include such factors as exercise, nutrition, social relationships, and social participation. The roof top components of the temple are socially or politically determined variables such as the adequacy of the social safety net, social support, and health care system. These three different layers of the components interact and compensate one another to determine longevity. In line with this conceptual framework, the Korean centenarian study has been carried out in a comprehensive manner with participation from multidisciplinary groups [6–9].

In this paper, we would like to focus on one of the pillars of our longevity model, that is, the nutritional characteristics of Korean centenarians. Vitamin B_12_ deficiency is a common nutritional deficiency among the elderly, particularly among the oldest old. Many of the oldest old suffer from atrophic gastritis, a thinning of the stomach lining that reduces the amount of B_12_ absorbed by the small intestine which may be related to Helicobacter pylori infection, pernicious anemia, and/or long-term ingestion of antacids or other medications. Surgery, digestive, and/or other medical conditions can also interfere with the absorption of this important micronutrient. Clinical manifestations are often subtle although they can be severe, particularly from a hematological or neuropsychiatric standpoint.

 One of the mysteries of Korean longevity has come from medical and nutritional assessment of centenarians that has indicated that many are relatively healthy, despite the lifelong traditional grain and vegetable-oriented dietary pattern. These findings are contradictory to the modern nutritional concept of nutritional balance for maintenance of health, since it is a challenge for most vegetarian (or semivegetarian) diets to supply adequate levels of several key nutrients, in particular vitamin B_12_. How Korean centenarians were able to avoid this serious age-related nutritional deficiency in spite of their low intake of animal products will be the focus of the this investigation.

## 2. Participants in Korean Centenarian Study

In our centenarian studies, age verification was prioritized. Since the civil registration system was not complete until the middle of 20th century in Korea, the age verification of the centenarians was processed by three different criteria including governmental registry, sibling age(s), and information from neighbors and acquaintances. Subjects who participated in our numerous centenarian studies [6–9] were randomly selected nationwide based on birth records, but those living in facilities like nursing homes or hospitals were excluded, because of the restriction in age verification due to lack of neighborhood information and family records. However, in Korea, there are generally fewer older people living in long-term care facilities compared to most developed nations and only 3.3% of people aging 65 or older are living in nursing homes or hospitals at present [10]. Unfortunately we do not have data on the exact numbers of centenarians in long-term care facilities, and it is well known that centenarians living in the community are generally higher functioning than those in long-term care facilities, therefore, we are likely dealing with a higher functioning sample population in our studies. 

The basic characteristics of subjects who participated in three centenarian studies are shown in Table 1. To our knowledge, the three cohorts were partially overlapping, though this is likely to be limited. 

The age range of centenarians was 100–108 years, and approximately 50% of male and 90% of female subjects were not educated at all, so that more than 80% were illiterate. Most of the centenarians were living with their family at home, and less than 10% of female centenarians were living alone. 

The average body weight and BMI were 52.3 kg and 22.2 kg/m^2^ in males and 34.4 kg and 17.6 kg/m^2^ in females, respectively [7].

## 3. Health Status and Blood Data of Korean Centenarians

Published data from three Korean centenarian surveys [6, 7, 9] on basic hematologic status and serum albumin, globulin, lipid, folate, vitamin B_12_ and homocysteine concentrations (nonfasting), were summarized in Table 2. Their results were similar to each other. The majority of these centenarian subjects were in relatively good health according to physical examinations and laboratory analyses. Ninety-five percent of the centenarian subjects had good appetites [6]. Kwak et al. [9] reported that average serum albumin concentration of Korean female centenarians was 3.75 g/dL, the prevalence of low serum albumin concentration (<3.5 g/dL) was 19.4%, and the average hemoglobin concentration and the anemic prevalence (<12.0 g/dL) were 11.3 g/dL and 56.7%, respectively, which were similar to the 12.1 g/dL and 50.2% found in Georgia centenarians [11]. 

Although Lee's study [6] has a limitation of small size regarding the number of male centenarians (only 6 subjects) and therefore caution should be exercised in making gender comparisons, it is nevertheless interesting to note that the RBC count and hematocrit level were significantly higher in male centenarians, and triglyceride and LDL-cholesterol levels were higher in females. Lee et al. [6] reported that none of the male centenarians were anemic, while 47.4% of female centenarians were mildly anemic (hemoglobin <11.2 g/dL), probably because of the higher consumption of protein and iron by male centenarians than female centenarians. Recently, Kwak et al. [9] reported that 56.7% of 62 Korean female centenarians were anemic (hemoglobin <12 g/dL). The Korean National Health and Nutritional Survey in 2005 (KNHNS) reported that the prevalence of anemia in older people aging 70 or more living in rural areas was 12.7% in males (hemoglobin <13 g/dL) and 14.5% in females (hemoglobin < 12.0 g/dL) [12].

 Some notable regional differences in health status were observed [8]. Only 4% of mountain-dwelling centenarians had serum albumin levels lower than 3.3 g/dL in contrast to 26% of seaside-dwelling centenarians. There was also a higher incidence of centenarians with anemia among seaside dwelling centenarians. These data may be showing a better health status for centenarians living in the mountains, which could be due to a higher level of physical activity and better supply of nutrients in quality and balance compared to seaside dwelling centenarians [8]. The higher HDL-cholesterol levels for males could be due to the differences in the levels of exercise and intakes of energy and protein. This supposition is supported by the abnormally low serum HDL-cholesterol levels in four-fifths of seaside dwelling centenarians compared to those who reside in mountainous areas.

## 4. Vitamin B_12_, Folate, and Homocysteine Status of Korean Centenarians

Risk factors for vitamin B_12_ deficiency include low animal protein intake, malabsorption associated with atrophic gastritis (which increases with age), or *Helicobacter pylori* infection, pancreatic or intestinal pathology, and gastric acid-reducing medications [13–16]. Poor vitamin B_12_ status has been associated with neurological problems [13, 17], hematological disorders [13, 18], and other health-related conditions, including poor cognition and Alzheimer's disease [19–21], depression [22], hearing loss [23], cancer [24], and poor bone health [25, 26]. More vegetarians or older people suffer from vitamin B_12_ deficiency compared to omnivores or younger adults [27]. Since natural sources of vitamin B_12_ in human diets are restricted for those who consume a diet low in foods of animal origin, vegetarians or semi-vegetarians are susceptible to cobalamin deficiency [28]. Moreover, the age-related increase of atrophic gastritis reduces production of gastric acid and digestive enzymes, required for cleavage of protein-bound vitamin B_12_ from the natural form of vitamin B_12_ in foods, which might aggravate vitamin B_12_ deficiency in the older people.

It is known that the prevalence of atrophic gastritis and *Helicobacter pylori* is very high in Korean adults. Yim et al. [29] reported that the seropositivity of *H. pylori* in asymptomatic health checkup adults nationwide in 2005 decreased to 59.6% from 66.9% in 1998. Other studies have also reported that 65.3% [30] and 56.2% [31] of adults who visited hospitals were *H. pylori* positive. 

Moreover, the prevalence of atrophic gastritis in antrum and body was reported to be 42.5% and 20.1%, respectively [30]. Therefore, it would be quite natural to assume that vitamin B_12_ status among Korean older people with lifelong habits of vegetable-oriented diets would be much worse when compared to that of older people in most Western societies where people tend to consume a diet much higher in foods of animal origin. 

Serum vitamin B_12_ and folate were measured by dual radioimmunoassay using ^57^Co/^125^I as a tracer, the most common method, with COBRA *Ⅱγ*-counter (Packard, UAS) by the lab at Eone Reference Laboratory [9] or Samsung Medical Center [6] in Korean centenarian studies. 

As shown in Table 2, average serum vitamin B_12_ concentration was 393.2 pg/mL and 405.5 pg/mL in male and female centenarians, respectively, in Lee's study [6], and 441.5 pg/mL in female centenarians in Kwak's study [9]. The prevalence of female centenarians with low serum vitamin B_12_ (<200 pg/mL) was 15.8% in Lee's study and 11.3% in Kwak's study, similar to the 11.6% found in American centenarians from Georgia [11]. 

It has been previously pointed out by numerous researchers that the standard cutoff points for serum vitamin B_12_ level (150 pmol/L, 200 pg/mL) are probably too low and may underestimate the frequency of true vitamin B_12_ deficiency in the population [32–34]; therefore, higher cutoff points (221–258 pmol/L, 300–350 pg/mL ) have been used for assessment of vitamin B_12_ deficiency in some surveys [11, 14, 32, 35]. Lindenbaum et al. [32] reported that 5.3% of the elderly group aging 67–96 years who participated in the Framingham study had serum vitamin B_12_ levels lower than 200 pg/mL, whereas 40.5% of the same elderly group and 12% of free-living elderly population had serum vitamin B_12_ levels lower than 350 pg/mL. It was reported that 33% of Italian centenarians had serum vitamin B_12_ levels lower than 300 pg/mL [35], and 39.1% of Georgia centenarians had serum vitamin B_12_ level lower than 340 pg/mL [11]. When assessed with a cutoff value of 340 pg/mL, the prevalence of vitamin B_12_ insufficiency in Korean female centenarians was 45.2% [9], similar to American centenarians from Georgia [11] and Italian centenarians [35]. These data strongly suggest that despite the traditional vegetable-heavy diet of older Koreans, there may still be good sources of vitamin B_12_ present within the diet. 

Metabolisms of vitamin B_12_, folate, and homocysteine are associated with and play very important roles in preventing many disorders of neurological and cognitive impairments as well as hematological dysfunctions in older people [36–38].

Dodge et al. [39] compared blood micronutrients among the oldest old (85 and over) in Okinawa and Oregon and reported that serum folate and vitamin B_12_ levels were negatively associated with serum homocysteine levels for the Okinawa cohort, who also had a very low usage of vitamin supplements in contrast to the Oregon cohort (who had a relatively high usage of vitamin supplements) and who showed no relationship among folate, vitamin B_12_, and homocysteine levels. 

 We have also analyzed associations among serum data for women aging 85 and over in a past study on elderly Koreans living in rural areas [9]. More specific data on multivitamin supplements were not collected, because few subjects of the cohort were taking vitamin supplements or functional foods, which are not commonly consumed among older people living in rural areas in Korea. We found that serum homocysteine concentration was not significantly correlated with age, serum folate, or vitamin B_12_; however, serum vitamin B_12_ was found to be positively associated with serum folate (*r* = 0.2266, *P* < .05) as well as WBC levels (*r* = 0.2623, *P* < .05), and serum folate was also positively correlated with RBC levels (*r* = 0.2685, *P* < .05) in this elderly cohort. Serum homocysteine levels were measured with automated chemiluminescence immune assay (CLIA) system in our study as was the case for the Dodge et al. study [39]. 

In Kwak's study [9], the average folate concentration in serum from non-fasting blood of Korean female centenarians was 5.79 ng/mL (13.1 nmol/L), and therefore within normal range, and the prevalence of folate deficiency (<3 ng/mL) was 33.8% [9]. Serum folate levels of Korean female centenarians were found to be much lower when compared with the 29.2 nmol/L levels of the American centenarians from Georgia [11], but similar to the 11.5 nmol/L levels found in Italian centenarians without cognitive impairment [35]. It was speculated that the reason for the lower serum folate levels in Korean centenarians when compared to those of Georgian centenarians might be related to a lower folate intake due to very low availability of folate-fortified foods in Korea in contrast to possible higher supplementation in the American oldest old. 

 The average serum homocysteine concentration of male centenarians was 24.9 *μ*mol/L and that of female centenarians was 21.2 *μ*mol/L in Kwon's study [7] and 22.3 *μ*mol/L in Kwak's study [9], showing no gender differences. These values were higher than the 14.5 *μ*mol/L of Georgian centenarians [11] but similar to that of cognitively intact Italian centenarians (22.0 *μ*mol/L) [35]. The prevalence of hyperhomocysteinemia (>17 *μ*mol/L) in Korean female centenarians was 73.0% [9], which was also similar to 77% of Italian centenarians with normal cognition [35], and 46.6% of Georgian centenarians were assessed to have hyperhomocysteinemia by lower criteria (>13.9 *μ*mol/L) [11]. To again exercise due caution in interpreting these results, it must be mentioned that different assay methods were used for measuring homocysteine (CLIA in Korean study, GC mass spectrometry in the Georgian study, and HPLC in the Italian study), so there may be some limitations when making these comparisons.

## 5. Food Intake, Variety, and Dietary Balance of Korean Centenarians

The Korean centenarian study [9] calculated the intake of dairy products, meat and eggs, fish and shellfish, cereals, potatoes and starch, sweets, legumes and tofu, vegetables and seaweeds, fruits, and soybean-fermented foods, as well as total food intake from a one-day dietary record (Table 3). 

The average total food intake of these female centenarians was 787.1 g/day. Meals were comprised primarily of plant foods (87.1% of total) such as cereals, legumes and their products, vegetables, and fruits. The average intake of cereals was 219.0 g/day, mostly derived from rice, a staple food for Koreans. The subjects consumed 29.7 g/day of legumes, nuts, and tofu, a representative soybean product consumed in Korea. They consumed 222.7 g/day of vegetables and seaweeds including 65.7 g/day of *Kimchi*, the most popular vegetable-fermented food in Korea, and a large portion of vegetable intake was derived from various blanched vegetables (*Namul* in Korean language). They also consumed 24.4 g/day of soybean-fermented foods, such *Doenjang (miso equivalent), Chungkookjang (natto equivalent), Gochujang (hot pepper paste), and Ganjang (soy bean sauce)*. Fruit intake was very low at 37.6 g/day, compared to vegetable intake. The subjects consumed 101.6 g/day of animal foods (12.9% of total), including 43.8 g of meat, poultry, and eggs, 37.6 g of fish and shellfish, and 18.1 g of dairy products. 

In addition, the dietary balance and variety of Korean centenarians' diet was evaluated using the dietary diversity score (DDS), the numbers of five food groups consumed in a day, and the dietary variety score (DVS), the number of different kinds of foods consumed in an entire day in two studies [6, 9]. The five groups and minimum amounts according to the DDS are (1) cereal and potatoes (≥60 g), (2) meat, fish, eggs, and their products (≥30 g), (3) milk and its products (≥60 mL/15 g in solid), (4) vegetables/vegetable juices (≥30 g/60 mL), and (5) fruits/fruit juices (≥30 g/60 mL) [40]. 

Looking at the results of Lee et al. [6], the average DDS was 3.33 in 6 male centenarians and 3.50 in 48 female centenarians, and the average DVS was 17.83 and 18.60, respectively (Table 3). Here, both DDS and DVS tended to be higher in female centenarians when compared to male centenarians, though not significantly so. Kwak et al. [9] reported that the average DDS and DVS of 74 female centenarians were 3.36 and 17.1, respectively. When assessed by the criteria for a well-balanced diet, which specify DDS > 3.0 and DVS > 18.0 [40, 41], 91.9% of these subjects scored above 3.0 in the DDS and 48.7% of subjects scored above 18.0 in the DVS (Table 3).

## 6. Energy and Nutrient Intake

Results of daily energy and nutrient intake of Korean centenarians [6, 9] are summarized in Table 4. Lee et al. [6] reported that the average energy intake was significantly higher in male centenarians when compared to female centenarians (1718 kcal/day versus 1247 kcal/day). Male centenarians consumed 85.9% of the estimated energy requirement (EER) for men aging 75 and over, 2000 kcal/day, and female centenarians consumed 77.9% of EER for women aging 75 and over, 1600 kcal/day [42]. EER for Korean men and women aging 75 and over was estimated on the reference body weight of 59.2 kg in men and 50.2 kg in women [42]. On comparing the average body weight of Korean centenarians, 52.3 kg in males and 34.4 kg in females [7], with the reference body weight of older people aging 75 and over, the body weight of female centenarians was much lower. The observed percentage of EER for energy intake in both Korean male and female centenarians was much higher when compared to the 60% found in a study of Okinawan centenarians [43]. 

Related with that higher energy intake, male centenarians consumed more protein and carbohydrate than female centenarians; however, fat intake in males and females was not different. Male centenarians consumed more calcium, zinc, vitamin B6, niacin, and vitamin E than female centenarians. 

Recently, Kwak et al. [9] reported that female centenarians consumed 1,186 kcal/day (74.1% of EER for the female elderly aging 75 and over) and 15.5% of total energy intake from protein, 13.9% from fat, and 70.6% from carbohydrate. They consumed 105.1% of the recommended intake (RI) of protein for the female elderly aging 75 and over, 45 g/day [42]. These female centenarians consumed 4.5 g dietary fiber, 123.7 mg cholesterol, 351.7 mg calcium (43.9% RI), 10.2 mg iron (113.3% RI), and 5.8 mg zinc daily (82.9% RI). In terms of vitamins, these subjects consumed 497.2 *μ*g RE of vitamin A (82.9% RI), 0.6 mg of vitamin B_1_ (54.5% RI), 1.3 mg of vitamin B_6_ (92.9% RI), 3.7 *μ*g of vitamin B_12_ (154.1% RI), 8.5 mg of niacin (60.7% RI), 47.6 mg of vitamin C (47.6% RI), 5.4 mg of vitamin E (54.0% AI, adequate intake), and 150.6 *μ*g of folate (37.6% RI). The average intake of fiber, calcium, niacin, and vitamins B_1_, C, and E was below 75% of the RI or AI for the respective nutrient. However, their nutrient intake levels might be underestimated, because the dietary reference intakes for the elderly aging 75 and over (not for centenarians) were used.

## 7. Analysis of Vitamin B_12_ Content in Korean Traditional Foods

Vitamin B_12_ is known to be synthesized only in certain bacteria [44]. The vitamin B_12_ synthesized by bacteria is concentrated mainly in the bodies of higher predatory organisms in the natural food chain system. Animal foods (i.e., meat, milk, egg, fish, and shellfish) have been considered to be the major dietary sources of vitamin B_12_. 

Surprisingly, the results of preliminary studies of centenarian diets showed that vitamin B_12_ status of Korean centenarians, who have consumed vegetable-based diets throughout their lives, was higher than our expectations. Therefore, we traced the unknown natural sources of vitamin B_12_ in traditional Korean foods [45]. Recently, for the first time, we reported the significantly high level of vitamin B_12_ content in some Korean traditional foods, soybean-fermented foods such as *Doenjang, Chungkookjang*, *Kochujang,* and *Ganjang* and vegetable-fermented foods such as Kimchi, and some favorite seaweed foods, that were not listed previously in Food Composition Tables [45]. The method for vitamin B_12_ assay in foods was the following: food samples were freeze-dried and then powdered. Total B_12_ was extracted by boiling at acidic pH range and assayed by the microbiological method with *L. delbrueckii* ATCC 7830 according to the method described by Watanabe et al. [46]. Since *L. delbrueckii* ATCC 7830 can utilize deoxyribosides and deoxyribonucleotides (known as an alkali-resistant factor) as well as B_12_, the amount of true B_12_ was calculated by subtracting the values of the alkali-resistant factor from the values of total B_12_. 

The key results were summarized in Table 5. It was interesting that vitamin B_12_ was not detected in steamed-soybeans and tofu; however, it was detected in fermented-soybean products. Moreover, traditional home-made soybean-fermented foods such as *Doenjang, Chungkookjang, *and* Gochujang* were found to contain higher vitamin B_12_ than commercial factory-made products. Traditional home-made *Doenjang* is a “slow food” taking at least 10 months for preparation and fermented by multiple microorganisms found in nature. However, the commercial product made in the factory takes only 3-4 months and is fermented by inoculated microorganisms under strict conditions. Due to the needs of space, time, and labor and the smell during the preparations and storage of *Doenjang*, the commercial *Doenjang* is increasingly popular, particularly to the younger generations living in urban areas. However, most Korean people living in rural areas still make it by themselves at home and consume it all year round. We observed that all the Korean centenarian subjects who participated in our studies were consuming the traditional home-made fermented foods. 

Most of Koreans consume *Kimchi*, a vegetable-fermented food, at almost every meal. There are a multitude of varieties of *Kimchi* in Korea, but *Cabbage Kimchi* is the most popular. It is made of salted Chinese cabbage, red pepper, garlic, fermented fish sauce or/and fermented small fish, green onion, ginger, starch, and some other optional vegetables and generally fermented for a few days, but sometimes for a few months in low temperature. It has been reported that the vitamin B_12_ content of *Kimchi* would be derived from the fermented fish sauce, one of the ingredients of *Kimchi *[45], rather than newly produced during the fermentation process. 

 Some kinds of edible seaweeds are traditionally consumed with flavoring by Koreans in fresh or dried and in raw or cooked forms. In particular, Koreans enjoy dried and toasted laver with salt and sesame oil or perilla oil.

## 8. Vitamin B_12_ Intake and Dietary Sources for Korean Centenarians

Generally, Korean centenarians do not consume supplements, and there are few vitamin B_12_-fortified foods in Korea. Only 3 out of 70 participants (4.3%) in a recent study [9] were found to be taking vitamin supplements.

We have updated the Korean vitamin B_12_ composition database [45] and have calculated daily dietary vitamin B_12_, intake of the female centenarian subjects using that updated database [9]. Total daily vitamin B_12_ intake and its dietary sources among the Korean traditional foods are identified and shown in Table 6. On average, these female centenarians consumed 3.73 *μ*g/day of vitamin B_12_ with 70.9% and 29.1% of total vitamin B_12_ intake derived from animal foods and plant foods, respectively. 

Korean centenarians were obtaining approximately 30% of their dietary vitamin B_12_ from foods of plant origin. In addition, although average daily vitamin B_12_ intake (3.73 *μ*g/day) of Korean centenarians was similar or less than that of female subjects aged 85 and older in Austria (3.9 *μ*g/day) or UK (4.3 *μ*g/day) [47], the prevalence of vitamin B_12_ deficiency in our cohort was not found to be higher when compared to cohorts in Western nations [9]. The primary food source of vitamin B_12_ was clearly meat, eggs, and fish, which provided two thirds of total vitamin B_12_ intake, and the next most popular food source was soybean-fermented foods, providing 13.9% of intake, followed by seaweeds, at 10.2%, and Kimchi and dairy products at 4.5% and 3.7%, respectively. 

 Korean centenarians have consumed soybean-fermented foods such as *Doenjang*, *Chungkukjang,* and *Gochujang* and fermented vegetables such as Kimchi daily as well as seaweeds very frequently, throughout their lives. Since these are consumed widely on a year-round basis, these foods represent very important sources of vitamin B_12_ for older Koreans. 

Some edible algae, including laver, have already been reported to contain large amounts of vitamin B_12_ [45, 48], though there are debates regarding the bioavailability of vitamin B_12_ in seaweeds [49–51]. However, the high consumption of dried seaweeds such as laver by Koreans would, nonetheless, still be partly responsible for the normal status of the vitamin B_12_ [52, 53].

The estimated average requirement (EAR) of vitamin B_12_ for elderly Koreans aging 75 and older is 2.0 *μ*g/day [42]. In order to find out how much foods of plant origin contributed to adequacy of vitamin B_12_ intake for Korean centenarians, we compared the adequacy of vitamin B_12_ intake from total foods to that from the animal foods. As shown in Table 7, the result from total food consumption showed that 51.4% of subjects consumed an adequate amount of vitamin B_12_ (above the EAR of 2.0 *μ*g/day) and 34.3% of subjects consumed a very low level of vitamin B_12_ (under 50% of the EAR), while the result from the analysis of animal foods showed that only 35.7% of subjects consumed an adequate amount of vitamin B_12_ while 42.9% of subjects consumed an inadequate amount of vitamin B_12_. These results imply that the consumption of Korean foods from plant sources, such as fermented foods and seaweeds, improved the nutritional status of vitamin B_12_ for these centenarians by increasing the percentage of the adequate vitamin B_12_ intake group by 15.7% and decreasing of the numbers of very low vitamin B_12_ intake group by 8.6%.

## 9. Summary and Conclusions

It is well known that most older Koreans traditionally consumed a diet low in animal foods and low in fat, dominated by cereals and vegetables. Centenarians in Korea seem to have been keeping to this traditional dietary pattern with one recent study revealing that female Korean centenarians were consuming 87.1% of the foods in their diet from plant sources [9]. 

Since major conventional food sources of vitamin B_12_ are well known to be of animal origins, we expected a higher prevalence of vitamin B_12_ deficiency in Korean centenarians compared to that found in centenarians in Europe or North America where consumption on animal products is much higher. However, the prevalence of Korean centenarians with a low serum vitamin B_12_ (<200 pg/mL) level was found to be only 11.3% and those with a marginal level of serum vitamin B_12_ (200–340 pg/mL) numbered only 33.9%. When assessed with a cutoff value of 340 pg/mL, the prevalence of vitamin B_12_ insufficiency in Korean female centeanrians was 45.2% [9], similar to American centeanrians from Georgia [11] and Italian centenarians [35].

When dealing with the mystery of why a much greater percentage of Korean centenarians did not suffer from vitamin B_12_ deficiency, we found that commonly consumed traditional Korean soybean-fermented foods (such as *Doenjang, Chungkukjang, and Ganjang*), vegetable-fermented foods with fermented fish sauce (such as *Kimchi*), and seaweeds (such as laver) contained higher than expected levels of vitamin B_12 _. Surprisingly, almost a third of vitamin B_12_ intake in the centenarian diet was coming from the consumption of these traditional foods.

These intriguing results from Korean centenarian studies suggest the value of a comprehensive, scientific approach in examining the traditional food culture and its potential contribution to maintaining an adequate nutritional status among the oldest old, as well as its potential contribution to healthy aging and longevity. 

As nutritional deficiency is an important contributor to the disease process and a particularly salient problem for the oldest old, new and economically viable solutions that focus upon improving nutritional status should be explored that are applicable (and potentially available) in cultural context. An excellent example is that of the complementary role that traditional foods have been playing in maintaining nutritional balance among centenarians in Korea.

## Figures and Tables

**Table 1:** Characteristics of Korean centenarian subjects.

	Lee et al. [6]	Kwon et al. [7]	Kwak et al. [9]
n(M/F)^†^	54 (6/48)	117 (13/104)	70 (0/70)

Age (years)	102.1 ± 1.7 (100–108)	102.2 ± 1.9 (100–108)	102.2 ± 1.9 (100–108)

Education (%)			
None	50.0/89.6^†^		/90.5^†^
Elementary	50.0/10.4		/9.5

Illiteracy (%)		86.3	

Smoking, currently (%)	19.6	40.0/17.4^†^	/25.7

Living arrangement (%)			
Alone	0.0/6.3^†^		/9.2
With family	100/93.7		/90.8
Only with spouse	16.7/0.0		/0.0

Weight (kg)			
M F		52.3 ± 3.9 34.4 ± 7.6	

BMI			
M F		22.2 ± 0.6 17.6 ± 3.6	

^†^Male/female.

**Table 2:** Blood biochemical variables including vitamin B_12_ level of Korean centenarians.^¶^

	Lee et al. [6]		Kwon et al. [7]		Kwak et al. [9]^†^
	Male (*n* = 6)	Female (*n* = 37)	Male (*n* = 13)	Female (*n* = 104)	Female (*n* = 62)
RBC (×100^3^/*μ*L)	4.0 ± 0.3*	3.6 ± 0.4			3.62 ± 0.66
WBC(×10^3^/*μ*L)	4.7 ± 1.7	4.5 ± 1.2			4.77 ± 1.71
Hemoglobin (g/dL)	12.8 ± 0.9	11.4 ± 1.3			11.3 ± 2.0
Anemic (Hb < 11.2)	0.0%	47.4%			
Anemic (Hb < 12)					56.7%
Hematocrit (%)	38.0 ± 2.7*	34.9 ± 3.7			34.7 ± 5.9
Albumin (g/dL)	3.7 ± 0.5	3.7 ± 0.4	3.7 ± 0.5	3.8 ± 0.4	3.75 ± 0.39
Low (<3.5)					19.4%
Globulin (g/dL)	3.3 ± 0.4	3.2 ± 0.5			3.16 ± 0.47
Triglyceride (mg/dL)	69.7 ± 20.6*	104.1 ± 59.3			103.4 ± 55.4
Total cholesterol (mg/dL)	155.2 ± 22.4	168.2 ± 36.9			168.7 ± 37.1
LDL cholesterol (mg/dL)	97.7 ± 9.8*	112.6 ± 32.7			110.8 ± 32.9
HDL cholesterol (mg/dL)	46.8 ± 16.9	42.5 ± 9.3			42.1 ± 9.4
Vitamin B_12_ (pg/mL)	393.2 ± 45.5	405.5 ± 26.4			441.5 ± 243.1
Deficient (<200)	0.0%	15.8%			11.3%
Marginal (≥200,<340)					33.9%
Adequate (≥340)					54.8%
Folate (ng/mL)	4.67 ± 4.24	5.67 ± 4.01			5.79 ± 3.80
Deficient (<3)	33.3%	28.9%			33.8%
Homocysteine (*μ*mol/L)	—	—	24.9 ± 9.3	21.1 ± 7.3	22.3 ± 7.6
Hyper (>17)			—	—	73.0%

Values are represented as mean ± SD.

^¶^All the parameters were analyzed in serum from notfasting blood samples.

^†^Data except vitamin B_12_ have not been published.

*Significantly different between males and females at *P* < .05.

**Table 3:** Daily food intake and dietary balance and variety in Korean centenarians.

	Lee et al. [6]	Kwak et al. [9]
Food intake (g)		787.1 ± 361.6 (100.0%)^†^
Plant (g)		685.4 ± 318.8 (87.1%)^†^
Animal (g)		101.6 ± 106.3 (12.9%)^†^

Cereals (g)		219.0 ± 80.0
Potatoes and starch (g)		14.3 ± 39.7
Sweets (g)		23.5 ± 29.5
Legumes, nuts & tofu (g)		29.7 ± 88.8
Vegetables & seaweeds (g) (Kimchi) (g)		222.7 ± 172.4 (65.7 ± 80.8)
Fruits (g)		80.8 ± 139.5
Soybean-fermented foods (g)		24.4 ± 30.0
Meat, poultry & eggs (g)		43.8 ± 51.1
Fish & shellfish (g)		37.6 ± 52.6
Dairy product (g)		18.1 ± 64.1

DDS	3.33 ± 0.62 (M) 3.50 ± 0.68 (F)	3.36 ± 0.73 (F) (91.9%)^‡^
DVS	17.83 ± 3.66 (M) 18.60 ± 5.69 (F)	17.1 ± 6.2 (F) (48.7%)^‡^

Values are expressed as means ± SD.

^†^% to total food intake.

^‡^% of subjects consuming well-balanced diet with higher score than 3.0, 18.0 in DDS or DVS.

DDS: dietary diversity score (0–5 points); DVS: dietary variety score.

**Table 4:** Daily energy and nutrient intake of Korean centenarians.

	Lee et al. [6]		Kwak et al. [9]
	Male (*n* = 6)	Female (*n* = 48)	Female (*n* = 70)
Energy (kcal)	1.718 ± 327**	1.247 ± 363	1.186 ± 418
Protein (g)	69.2 ± 25.6**	40.8 ± 18.4	47.3 ± 21.7 (15.5%)^†^
Fat (g)	27.0 ± 8.7	19.3 ± 12.3	19.8 ± 12.4 (13.9%)^†^
Carbohydrate (g)	295.3 ± 67.9*	225.9 ± 65.1	215.6 ± 72.1 (70.6%)^†^
Fiber (g)	6.8 ± 4.45	5.0 ± 3.2	4.5 ± 2.9
Cholesterol (mg)	269.0 ± 259.4	115.8 ± 161.2	123.7 ± 159.3
Calcium (mg)	564.1 ± 237.9*	352.7 ± 202.8	351.7 ± 193.6
Iron (mg)	12.9 ± 4.1	8.90 ± 5.02	10.2 ± 5.9
Zinc (mg)	9.21 ± 3.76**	5.86 ± 2.31	5.8 ± 2.8
Vitamin A (RE)	878.9 ± 600.7	586.1 ± 438.7	497.2 ± 424.8
Vitamin B_1_ (mg)	1.0 ± 0.2	0.7 ± 0.4	0.6 ± 0.3
Vitamin B_2_ (*mg) *	0.9 ± 0.3	0.7 ± 0.4	—
Vitamin B_6_ (mg)	2.0 ± 0.5**	1.3 ± 0.6	1.3 ± 0.6
Vitamin B_12_ (*μ*g)	—	—	3.7 ± 5.7
Niacin (mg)	15.8 ± 5.8***	8.9 ± 4.4	8.5 ± 4.2
Vitamin C (mg)	72.8 ± 59.5	55.9 ± 39.0	47.6 ± 32.4
Vitamin E (mg)	10.7 ± 6.7*	5.8 ± 4.3	5.4 ± 4.3
Folate (*μ*g)	—	—	150.6 ± 92.3

Values are represented as mean ± SD.

Significantly different between males and females at *P < .05, **P < .01, or ***P < .001.

^†^% to total calorie intake.

**Table 5:** Vitamin B_12_ content in Korean fermented foods and some popular foods.^†^

		
Food	Vitamin B_12_ content^(1)^	
	(*μ*g/100 g dry wt)	(*μ*g/100 g wet wt)
*Soybean*, steamed	0.00	
*Tofu*	0.00	
*Doenjang*		
Traditional, home-made (*n* = 30)	0.30 ~ 9.82^(2)^	0.14 ~ 4.41^(3)^
Commercial, factory-made (*n* = 4)	0.07 ~ 0.49	0.04 ~ 0.25
*Chungkookjang*		
Traditional, home-made (*n* = 5)	0.05 ~ 1.40	0.03 ~ 0.60
Commercial, factory-made (*n* = 3)	0.08 ~ 0.31	0.04 ~ 0.15
*Gochujang*		
Traditional, homemade (*n* = 10)	0.02 ~ 0.43	0.01 ~ 0.28
Commercial, factory-made (*n* = 3)	0.00 ~ 0.14	0.00 ~ 0.01
*Ganjang (Soy sauce)*		(*μ*g/100 mL)
Korean-style, homemade (*n* = 29)		0.02 ~ 6.76
Japanese-style, commercial (*n* = 4)		0.00
*Fish sauce*		(*μ*g/100 mL)
Shrimp, salt-fermented (*n* = 2)		0.78 ~ 0.91
Anchovy, salt-fermented (*n* = 2)		1.52 ~ 1.77
*Kimchi*		
Korean Cabbage Kimchi (*n* = 3)	0.18 ~ 0.24	0.18 ~ 0.22
*Seaweeds*		
Laver, dried, seasoned & toasted (*n* = 3)	55.3 ~ 71.3	
Sea lettuce, raw (*n* = 1)	84.7	9.41
Sea tangle, dried (*n* = 1)	0.36	
Sea mustard, dried (*n* = 1)	1.90	
*Anchovy*,		
dried, medium size (*n* = 1)	17.12	

^†^Summary of key results from a report by Kwak et al. [45] and new data.

(1) Vitamin B_12_ = total vitamin B_12_ − alkali resistant factor.

(2) Range of vitamin B_12_ contents in more than two different products.

(3) Calculated from average vitamin B_12_ content measured in dried sample and drying yield.

**Table 6:** Daily mean intake and dietary source of vitamin B_12_ of female Korean centenarians.

	Kwak et al. (2010) [9]
	Female (*n* = 70)
Meat, eggs, fish & shell (*μ*g)	3.04 ± 5.69 (67.2%)^†^
Dairy products (*μ*g)	0.05 ± 0.19 (3.7%)
Animal (*μ*g)	3.09 ± 5.68 (70.9%)

Kimchi (*μ*g)	0.02 ± 0.02 (4.5%)
Soybean-fermented foods (*μ*g)	0.08 ± 0.16 (13.9%)
Seaweeds (*μ*g)	0.53 ± 1.37 (10.2%)
Others (*μ*g)	0.01 ± 0.01 (0.5%)
Plant (*μ*g)	0.64 ± 1.36 (29.1%)

Total B_12_ intake (*μ*g)	3.73 ± 5.79 (100.0%)

Values are represented as mean ± SD.

^†^Mean of percent to total vitamin B_12 _ intake.

**Table 7:** Distribution of daily vitamin B_12_ intake from total and animal foods.

		
	from total food^(1)^ *n* (%)	from animal food^(2)^ *n* (%)
*B_12_ intake (*μ*g/day)*		
Deficient <1.0	24 (34.3)	30 (42.9)
≥1.0 and <2.0	10 (14.3)	15 (21.4)
Adequate ≥2.0^(3)^	36 (51.4)	25 (35.7)

	70 (100.0)	70 (100.0)

(1) Reference Kwak et al. [9].

(2) Newly analyzed.

(3) EAR of vitamin B_12_ for Korean older people aged 75 years and more.
